# Purification and characterization of the first γ-phospholipase inhibitor (γPLI) from *Bothrops jararaca* snake serum

**DOI:** 10.1371/journal.pone.0193105

**Published:** 2018-03-05

**Authors:** Caroline Serino-Silva, Karen Morais-Zani, Marcos Hikari Toyama, Daniela de Oliveira Toyama, Henrique Hessel Gaeta, Caroline Fabri Bittencourt Rodrigues, Wéslei da Silva Aguiar, Alexandre Keiji Tashima, Kathleen Fernandes Grego, Anita Mitico Tanaka-Azevedo

**Affiliations:** 1 Interunidades em Biotecnologia, Universidade de São Paulo – Instituto de Pesquisas Tecnológicas – Instituto Butantan, São Paulo, São Paulo, Brasil; 2 Laboratório de Herpetologia, Instituto Butantan, São Paulo, São Paulo, Brasil; 3 Instituto de Biociências do Litoral Paulista, Universidade Estadual Paulista, São Vicente, São Paulo, Brasil; 4 Departamento de Bioquímica, Universidade Federal de São Paulo, São Paulo, São Paulo, Brasil; Universidad de Costa Rica, COSTA RICA

## Abstract

Phospholipases A_2_ (PLA_2_) are enzymes acting on the cell membrane phospholipids resulting in fatty acids and lysophospholipids and deconstructing the cell membrane. This protein is commonly found in snake venoms, causing tissue inflammation in the affected area. Evidence indicates that snakes have natural resistance to their own venom due to protective properties in plasma, that inhibit the action of proteins present in their venom. Given that, this study aimed to purify and characterize a γPLI from *Bothrops jararaca* serum, named γBjPLI. PLA_2_ inhibitor was isolated using two chromatographic steps: an ion exchange column (DEAE), followed by an affinity column (crotoxin coupled to a CNBr-activated Sepharose resin). The purity and biochemical characterization of the isolated protein were analyzed by RP-HPLC, SEC, SDS-PAGE, circular dichroism and mass spectrometry. The ability to inhibit PLA_2_ was determined by enzymatic activity, neutralization of paw edema and myonecrosis. The protein purity was confirmed by RP-HPLC and SEC, whilst an apparent molecular mass of 25 kDa and 20 kDa was obtained by SDS-PAGE, under reducing and non-reducing conditions, respectively. According to mass spectrometry analysis, this protein showed 72% and 68% of coverage when aligned to amino acid sequences of two proteins already described as PLIs. Thus, the inhibitory activity of enzymatic, edema and myonecrotic activities by γBjPLI suggests a role of this inhibitor for protection of these snakes against self-envenomation.

## 1. Introduction

Snakebite envenoming is classified as a neglected tropical disease by the World Health Organization [1]. *Bothrops* genus (Viperidae family) is responsible for ~86% of snake accidents in Southeastern Brazil, according to recent epidemiological data from Brazilian Ministry of Health [2,3]. Snake venoms have a high diversity of proteins and peptides, which display a wide repertoire of pharmacological and toxic actions [4–6]. Clinically, patients bitten by *Bothrops sp*. usually present local effects (pain, edema, myonecrosis, local hemorrhage, blistering, and tissue necrosis) and systemic damage (coagulopathy, systemic hemorrhage, cardiovascular shock and acute kidney injury) [7–14]. The antivenom therapy is highly effective concerning neutralization of toxins which cause systemic effects, but is not efficient to neutralize local damages caused mainly by the action of metalloproteinases (EC 3.4) and phospholipases A_2_ (PLA_2_, EC 3.1.1.4) [15].

PLA_2_ specifically hydrolyzes the *sn-2* ester bond of phospholipids to produce lysophospholipids and fatty acids. Among its by-products it is found arachidonic acid, a precursor of the inflammation cascade [16,17]. The PLA_2_s are one of the most abundant toxins in the Viperidae family [18,19], being widely distributed in living organisms, including mammals. The snake venom PLA_2_s are secreted-type small proteins (12–15 kDa) composed by 119–134 amino acids and, according to structural and biochemical properties, these proteins are classified in Group I (Elapidae) or Group II (Viperidae) PLA_2_s (I–XV), differing only in the position of disulfide bond and in C-terminal regions [20]. Group II can be subdivided in two other subgroups, Asp49 PLA_2_s and PLA_2_ homologues. The last subgroup presents an amino acid substitution, where Asp in its catalytic center (49 position) is substituted by another amino acid (Lys, or less frequently by Ser, Arg, Gln or Asn). This change in catalytic site is responsible for the loss of ability to bind to Ca^2+^ as a cofactor, reducing or fully precluding the enzymatic activity, however the protein still remains extremely active in the induction of myonecrosis [21,22]. Snake venom PLA_2_s can also induce several effects, such as pre- or postsynaptic neurotoxicity, cardiotoxicity, platelet aggregation inhibition or induction, edema, hemolysis, anticoagulation, convulsion and hypotension [23–26].

A large number of biological compounds found in plasma of some animals capable of inhibiting the snake venoms actions are already known, including proteins in the plasma of some mammals as the Virginian opossum (*Didelphis virginiana* [27,28]), the Indian grey mongoose (*Herpestes edwardsii* [29]) and the European hedgehog (*Erinaceus europaeus* [30]); in the plasma of some venomous snakes as the Brazilian lancehead (*Bothrops moojeni* [31]), the jararacussu (*Bothrops jararacussu* [25]), other *Bothrops* sp snakes (*B*. *alternatus*, *B*. *erythromelas* and *B*. *neuwiedi* [32]), the rattlesnake (*Crotalus durissus terrificus* [33] and *Crotalus durissus collineatus* [34]), the bushmaster snake (*Lachesis muta muta* [35]), the coral snake (*Micrurus lemniscatus* [36]) and the habu snake (*Trimeresurus flavoviridis* [37]); as well as in the plasma of some non-venomous snakes as the Akamata (*Dinodon semicarinatus* [38]), the Japanese striped snake (*Elaphe quadrivirgata* [39,40]) and the Japanese rat snake (*Elaphe climacophora* [41]).

The inhibitors of PLA_2_ (PLI) have been intensely studied, and are classified in three types (α, β and γ) based on the presence of characteristic structural domains, which can be concomitantly found in a single specimen [42,43]. γPLI has 30–90 kDa, with 3–6 non-covalent subunits of 15–31 kDa, which are composed of highly conserved structural units of two tandem cysteine repeats, characteristic of the three-finger motifs [42]. Although several PLIs have been identified and their sequences have been described through molecular techniques (such as cDNA sequencing), studies showing the isolation and characterization of these molecules from snake and other animals serum or plasma are scarce [32,35,36,44].

In this context, the present study reports for the first time, to the best of our knowledge, the isolation and characterization of a γPLI from jararaca (*Bothrops jararaca*) **s**erum, the most common species in the southeast region of Brazil and accounting for the majority of accidents in this area [45].

## 2. Material and methods

### 2.1. Ethics statement

The Laboratory of Herpetology of Butantan Institute, São Paulo (Brazil), supplied blood of *B*. *jararaca* and venom of *Crotalus durissus terrificus* (*C*. *d*. *terrificus*). The Animal House of Butantan Institute, São Paulo (Brazil) supplied specimens of Swiss mice. The ethical Committee for the Use of Animals of Butantan Institute approved these experimental protocols (1374/15 CEUAIB). After the experiments, the animals (female Swiss mice) were sacrificed with CO_2_. This proposal is in accordance with standards outlined by Brazilian laws for use of experimental animals, and with ethical principles adopted by the Brazilian College of Animal Experimentation (COBEA).

### 2.2. *B*. *jararaca* blood collection and serum separation

*B*. *jararaca* blood (10 specimens) was collected by caudal venipuncture and maintained at 4°C overnight. Afterwards, the serum was obtained by centrifugation at 1200 × g for 15 min at 4°C, and stored at -20°C.

### 2.3. *C*. *d*. *terrificus* venom

Pooled lyophilized venoms of *C*. *d*. *terrificus* were supplied by the Laboratory of Herpetology at Butantan Institute, São Paulo (Brazil).

### 2.4. Purification of the crotoxin and PLA_2_

Crotoxin was purified by fractionation of *C*. *d*. *terrificus* venom by size-exclusion chromatography, using a Sephacryl S200 HR column. Lyophilized crude venom pool of *C*. *d*. *terrificus* snakes (449 mg), was dissolved in 2.5 mL of 50 mM Tris, 0.1 M NaCl buffer (pH 7.4), centrifuged at 4500 × g for 15 min at 4°C and filtered by 0.45 microfilter. The supernatant was applied on the column, previously equilibrated with 50 mM Tris, 0.1 M NaCl, pH 7.4. The PLA_2_ was separated from crotapotin by Reverse Phase-High Performance Chromatography (RP-HPLC) using a Supelco C5 column (0.10 cm × 25 cm), as previously described by Toyama et al. [46].

### 2.5. Purification of the γBjPLI

γBjPLI was isolated from *B*. *jararaca* serum by a combination of two chromatographic steps: an anion exchange and afterwards an affinity chromatography. The purity of the isolated molecule was evaluated by RP-HPLC on C18 column.

#### Anionic exchange chromatography

Seven milliliters of *B*. *jararaca* serum were diluted in 7 mL of 25 mM Tris buffer, pH 7.5 (buffer A) and injected onto a DEAE Fast Flow column (1.6 x 2.5 cm) (GE Healthcare), previously equilibrated with 95% of buffer A and 5% of buffer B (25 mM Tris, 1 M NaCl, pH 7.5). The elution was performed by two steps, initially using buffer A containing 100 mM NaCl, followed by a linear gradient of 100 mM to 500 mM NaCl. The last eluted peak (named D2) was dialyzed three times against PBS (using a membrane with a 12 kDa cut-off molecular weigth) during 16 h at 4°C.

#### Affinity chromatography

Crotoxin (20 mg), isolated from *C*. *d*. *terrificus* venom as described in item 2.4, was coupled to 2 g of CNBr-activated Sepharose as described by the manufacturer (GE Healthcare). At the end of the coupling process, a wash sequence is made with acidic (pH 4) and basic (pH 8.3) buffers to remove molecules that would not be bound to resin correctly. It was then settled in a glass column and equilibrated with PBS. A control chromatography without serum sample was performed, in order to verify if crotoxin molecules were being detached from the resin. For the second purification step of γBjPLI, D2 peak (from anionic exchange) was applied on the affinity column (crotoxin + CNBr-activated Sepharose). The column was washed with PBS again, and the inhibitor was eluted with 1 M glycine, pH 2. Fractions of 2 mL were collected, and the pH was immediately neutralized with 1 M Tris buffer, pH 8.8.

### 2.6. RP-HPLC

γBjPLI purity was analyzed using RP-HPLC on C18 column (Discovery BIO Wide Pore C18 HPLC Column, 25 cm × 4.6 mm). In this chromatography, approximately 1 mg of γBjPLI was dissolved in solution A (TFA 0.1%) that was also used for RP-HPLC equilibration for 15 min, before injection of samples. The elution of all samples was done using a linear gradient (0–100%) of solution B (66% ACN in solution A) and chromatographic run was conducted at 280 nm, at a constant flow rate of 1 mL/min for 40 minutes.

### 2.7. Size exclusion chromatography protein analysis (SEC)

In order to perform the protein analysis by SEC, samples of isolated PLA_2_, purified γBjPLI and PLA_2_ incubated with γBjPLI (PLA_2_ + γBjPLI) were prepared by dissolution of each protein in 0.05 M Tris-HCl buffer, pH 7.6 at a final protein concentration of 1 mg/mL. The chromatographic system composed by analytical SEC column separation was performed on silica-based column BioSep SEC S-2000 from Phenomenex (5 μm, 300 × 7.8 mm), maintained at 30°C, coupled to analytical chromatography system Jasco. The chromatographic column was equilibrated with 0.05 M Tris-HCl buffer, pH 7.6 for 20 min before injection of 10 μL of each sample. The chromatographic run was monitored at 280 nm, at a flow rate of 1 mL/min and all fractions were recovered, lyophilized and stored for further investigations. For size comparisons, a Gel Filtration Standart (BioRad) was used, at the ranges from 670 kDa to 1.35 kDa.

### 2.8. Sodium Dodecyl Sulfate Polyacrylamide Gel Electrophoresis (SDS-PAGE)

SDS-PAGE using 15% polyacrylamide gels was performed according to Laemmli [47], in the presence or absence of reducing agent (β-mercaptoethanol). Gels were electrophoresed at constant amperage of 20 mA and the samples were stained with Coomassie brilliant blue G-250.

### 2.9. Mass spectrometry

Protein bands were excised from SDS-PAGE and subjected to reduction (10 mM dithiothreitol), alkylation (50 mM iodoacetamide), and overnight in-gel digestion with sequencing grade trypsin (Sigma), in 50 mM ammonium bicarbonate at 37°C. Tryptic peptides were extracted with 1% acid formic and analyzed by nanoAcquity UPLC, coupled to a Synapt G2 HDMS mass spectrometer (Waters). The ion source was made by ESI (nano-spray), fragmentation by CID and CAD (y and b ions), MS scan by Quadrupole and MS/MS scan by Time of Flight (TOF). Fragment mass error tolerance was of 0.1 Da. A peak list was generated, and used to search against the “Uniprot_serpentes_unr” database. The alignment of the amino acid sequence obtained by mass spectrometry analysis was done using the program Clustal Omega 1.2.4 [48].

### 2.10. Circular dichroism spectroscopy (CD)

PLA_2_ and γBjPLI were dissolved in 10 mM sodium phosphate buffer (pH 7.4) and the final protein concentration was adjusted to 2.15 mM. After centrifugation at 4000 × g for 5 min, samples were transferred to a 1 mm path-length quartz cuvette. Circular dichroism (CD) spectra in the “far UV” region (185–260 nm) was acquired with a J815 spectropolarimeter (Jasco Corp.) using a bandwidth of 1 nm and a time response of 1 s. Data collection was performed at room temperature with a scanning speed of 100 nm/min. Nine scans were obtained for each sample, and all spectra were corrected by subtracting the buffer blanks.

### 2.11. Measurement of PLA_2_ activity and the inhibitory effect of γBjPLI upon PLA_2_

PLA_2_ activity was measured following the protocol described by Holzer and Mackessy [49] for 96-wells plate assay, using 4-nitro-3-octanoyloxy-benzoic acid (4N3OBA, Enzy Life Science) as substrate. The standard assay mixture contained 200 μL of buffer (10 mM Tris/HCl, 10 mM CaCl_2_, and 100 mM NaCl, pH 7.8), 20 μL of substrate, 20 μL of pure water and 20 μL of PLA_2_ sample (20 μg) and were incubated for up to 40 min at 37°C. The hydrolysis values were determined by measuring the absorbance at 405 nm at 5-min intervals. The γBjPLI inhibitory effect upon PLA_2_ was determined by measuring the increase of absorbance after a previously incubation of the PLA_2_ (20 μg) and γBjPLI (20 μg, 10 μg, 5 μg or 2,5 μg of γBjPLI) mixture for 20 min at 37°C. All assays were performed in triplicate, and the absorbance at 405 nm was measured using a SpectraMax 340 multiwell plate reader (Molecular Devices). The absorbances were transformed to velocity of substrate consumption (nmol/min). The inhibition percentage was determined by linear regression through ranges of 20 to 30 minutes. The rate of substrate consumption (slope of the line) was calculated using the formula ((VoPLA2—VoγBjPLI) / VoPLA2) * 100.

### 2.12. Paw edema inhibition by γBjPLI

The γBjPLI role on edema development was analyzed by paw edema, which was induced by a single sub plantar injection in female Swiss mice (~25 g) of 10 μg of PLA_2_ that was previously incubated for 30 min with 10 μg of γBjPLI (n = 5). The control groups were inoculated with 10 μg of γBjPLI (n = 5), saline (0.9%) (n = 5) or 10 μg of PLA_2_ (n = 5). The paw volumes were measured immediately before the injection and at selected time intervals thereafter (0, 30, 60, 90, 120, 180, 240 and 300 minutes) using a hydroplethysmometer (model 7150, Ugo Basile). The results were expressed as the increase in paw volume (μL) calculated by subtracting the initial volume (0 min) from the final volume.

### 2.13. Evaluation of myonecrosis inhibition by γBjPLI

The myonecrosis inhibition by γBjPLI was determined by the injection of 20 μL of PLA_2_ (20 μg) incubated for 30 min with γBjPLI (20 μg) (n = 5) on the right *gastrocnemius* muscle (21 g female Swiss mice). Control groups received 20 μg of PLA_2_ (n = 5) or 20 μg of γBjPLI (n = 5) or 20 μL of 0.9% saline (n = 5). Mice blood samples were collected into tubes containing heparin as anticoagulant, centrifuged and the plasma was separated and stored at 4°C for a maximum of 12 h before the assay. The amount of Creatine Kinase (CK) was then determined using 40 μL of plasma, which was incubated for 3 minutes at 37°C with 1 mL of the reagent according to the kit protocol of CK commercial kit (Bioliquid). The resulting activity was expressed in U/L.

### 2.14. Statistical analysis

Values are expressed as the mean ± S.E.M. For statistical analyses, one-way analysis of variance (ANOVA) followed by the Student–Newman–Keuls *post hoc* test was used to analyze CK tests. Two-way ANOVA followed by Bonferroni *post hoc* test was used to analyze paw edema. The results were considered statistically significant at probability (P) values equal to or less than 0.05.

## 3. Results

### 3.1. Purification of the inhibitor γBjPLI

**Fig 1:**
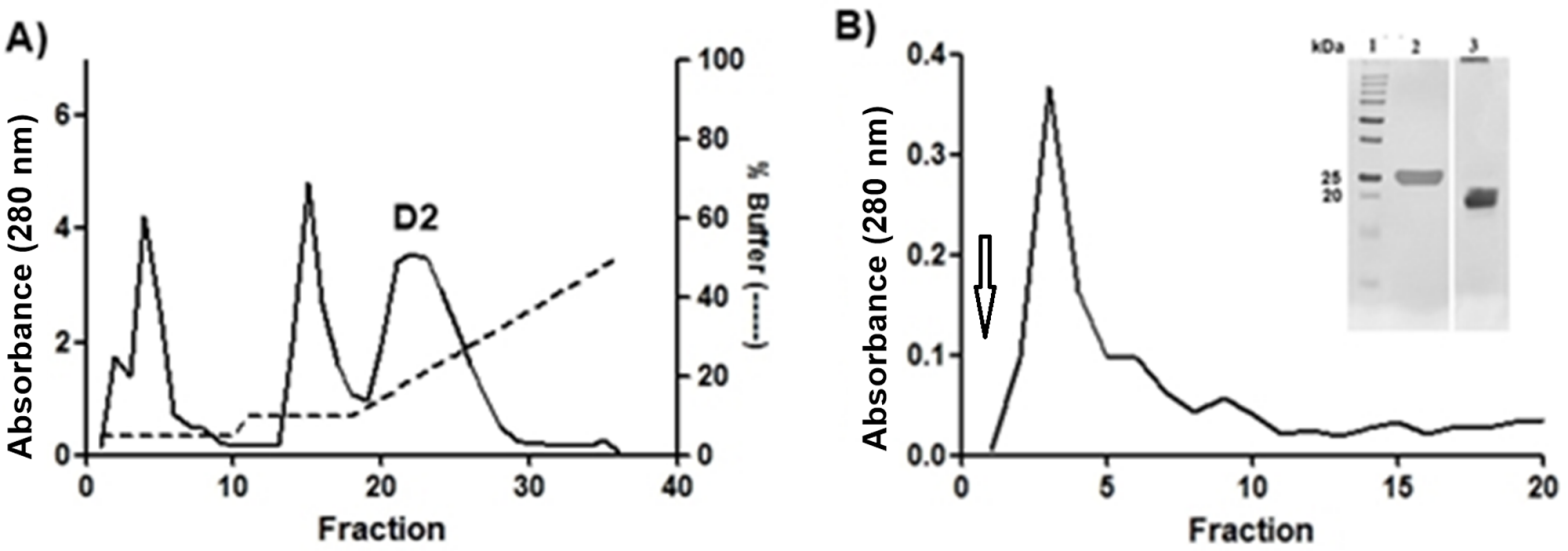
Purification of γBjPLI. A) Fractionation of *B*. *jararaca* serum on ion exchange column (DEAE). Elution was initially done keeping buffer A (25 mM Tris, pH 7.5), 10% of buffer B (25 mM Tris, 1M NaCl pH 7.5) and then making a gradient of B up to 50% (500 mM NaCl), with a flow rate of 1 mL/min. B) Elution of the fractions of pool D2 applied to an affinity column (CNBr-activated Sepharose + crotoxin), done with 1 M glycine pH 2 (indicated by an arrow). SDS-PAGE: 1. Molecular mass marker (Dual Color Precision Plus, BioRad) 2. γBjPLI with β-mercaptoethanol; 3. γBjPLI without β-mercaptoethanol.

The γBjPLI was purified by two chromatographic steps, using an ion exchange column (DEAE) (Fig 1A), followed by an affinity column (crotoxin coupled to a CNBr-activated Sepharose resin) (Fig 1B). According to the purification table the eluted fraction of affinity chromatography represents 1% of the total protein of the snake serum (Table 1).

**Table 1:** Purification of γBjPLI from *B*. *jararaca* serum.

Sample	Protein content (mg)* [50]	Recovery (%)
Serum	218	100
Ion exchange column (DEAE)	103.57	47
Affinity column (CNBr-activated Sepharose resin + crotoxin)	2.18	1

*Quantified using the Bradford reagent and bovine serum albumin (BSA) as standard.

**Fig 2:**
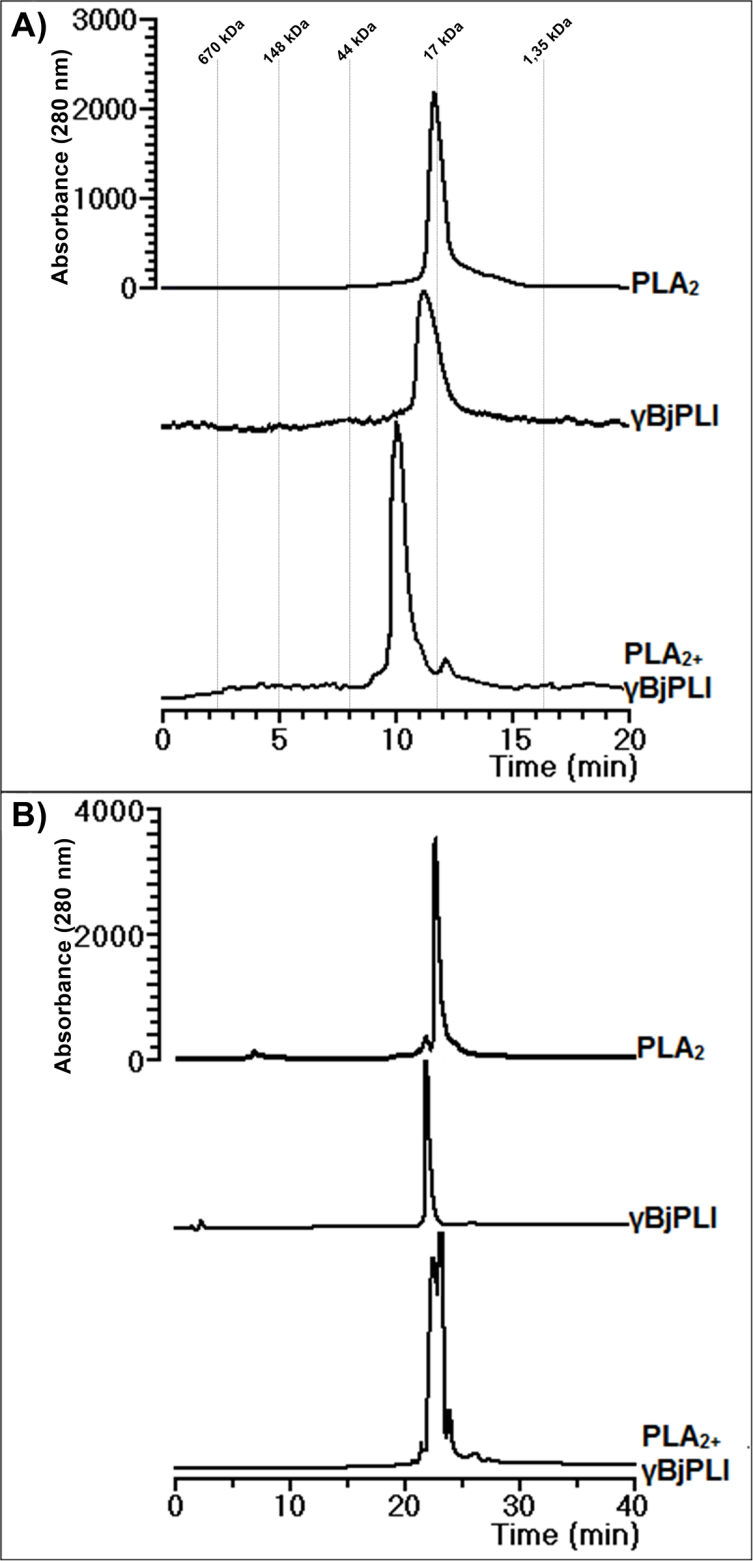
Interaction analysis on chromatographic columns. A) Chromatographic profile on BioSep SEC S-2000 of PLA_2_ (500 μg) and γBjPLI (500 μg) samples applied to the column alone or preincubated together. The run was performed in 0.05 M tris-HCl buffer, pH 7.6 at a final protein concentration of 1 mg/mL, at a flow rate of 1 mL/min. B) Chromatographic profile on C18 column (RP-HPLC) of PLA_2_ (1 mg) and γBjPLI (1 mg) samples applied to the column alone or preincubated together. The run was performed in 0.1% TFA (solution A) and 66% acetonitrile and 0.1% TFA (solution B) in a linear gradient, at a flow rate of 1 mL/min. All analyses were monitored at 280 nm.

The electrophoretic profile of the sample eluted from the affinity chromatography was analyzed through SDS-PAGE, and showed a single band around 20 kDa in non-reduced condition, but in reduced condition this major band presented around 25 kDa, as shown in Fig 1. The γBjPLI purity was evaluated by RP-HPLC on C18 (Fig 2B).

### 3.2. Size exclusion chromatography protein analysis

SEC analysis showed that γBjPLI appears as a single fraction, as well as PLA_2_. Furthermore, it is possible to observe an interaction between PLA_2_ and γBjPLI, when both molecules are pre-incubated together, through a change in the retention time profile, presenting an estimated molecular mass of approximately 35 kDa (Fig 2A). However, this complex cannot be observed by RP-HPLC (Fig 2B).

### 3.3. Mass spectrometry

Protein band corresponding to γBjPLI was excised from SDS-PAGE, digested with trypsin and analyzed by mass spectrometry, presenting 72%, 68% and 41% coverage of the amino acid sequence of proteins described as γPLI, deposited in the transcriptomic database (Table 2). The two highest coverage proteins were identified as γPLI from *B*. *jararaca*, followed by *Bothrops moojeni*.

**Table 2:** Proteins identified with higher percentage of coverage from the peptides obtained from the band excised from SDS-PAGE and analyzed by mass spectrometry in Synapt G2 HDMS (Waters).

Protein	Coverage	Description	Mass (Da)	Peptides*
A8I4L6|A8I4L6_BOTJA	72%	γPLI *Bothrops jararaca*	22 070	19
A8I4M0|A8I4M0_BOTJA	68%	γPLI *Bothrops jararaca*	22 197	19
A8I4N5|A8I4N5_BOTMO	41%	γPLI *Bothrops moojeni*	22 181	12

* The complete peptide table is available as supplemental data.

### 3.4. Circular dichroism analysis

**Fig 3:**
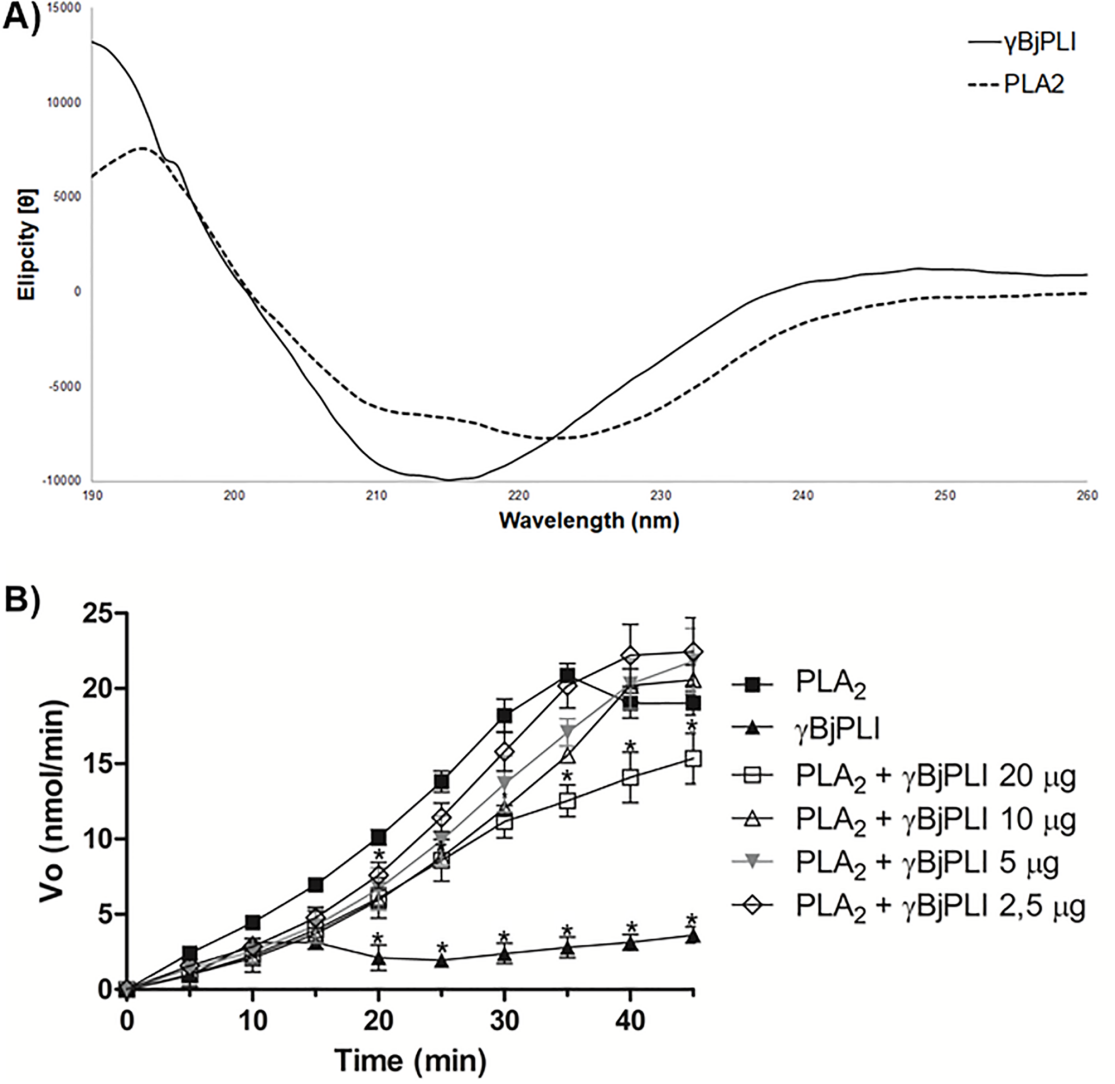
γBjPLI and PLA_2_ secondary structure and enzymatic activity (*in vitro*). A) γBjPLI and PLA_2_ spectrum obtained by circular dichroism analysis for wavelengths between 190 and 260 nm. The data was expressed in molar elipticity. B) Enzyme activity inhibition test of PLA_2_ by γBjPLI. Different doses of γBjPLI (20 μg, 10 μg, 5 μg, 2,5 μg) and fixed dose of PLA_2_ (20 μg) were used. The γBjPLI + PLA_2_ samples were incubated for 40 min at 37°C. Substrate 4N3OBA (1 mg/mL) was then added and monitored at 405 nm for 40 min and the data was expressed in nmol/min.

γBjPLI was subjected to CD and showed the presence of many alpha-helices (26.3%) and random structures (34.5%), followed by pleated beta sheets (18.5%). In comparison, isolated PLA_2_ showed approximately 24.1% of alpha-helices and 39.3% of random structures (Fig 3A).

### 3.5. Enzymatic assay

To confirm that the γBjPLI was able to inhibit PLA_2_, an *in vitro* test was initially performed. The results showed that γBjPLI was capable of inhibiting PLA_2_ in a dose-dependent manner. The highest percentage was 40% of inhibition when 20 μg for γBjPLI was used (1:1 molar ratio; γBjPLI:PLA_2_) (Fig 3B).

### 3.6. Paw edema and myonecrosis evaluation

**Fig 4:**
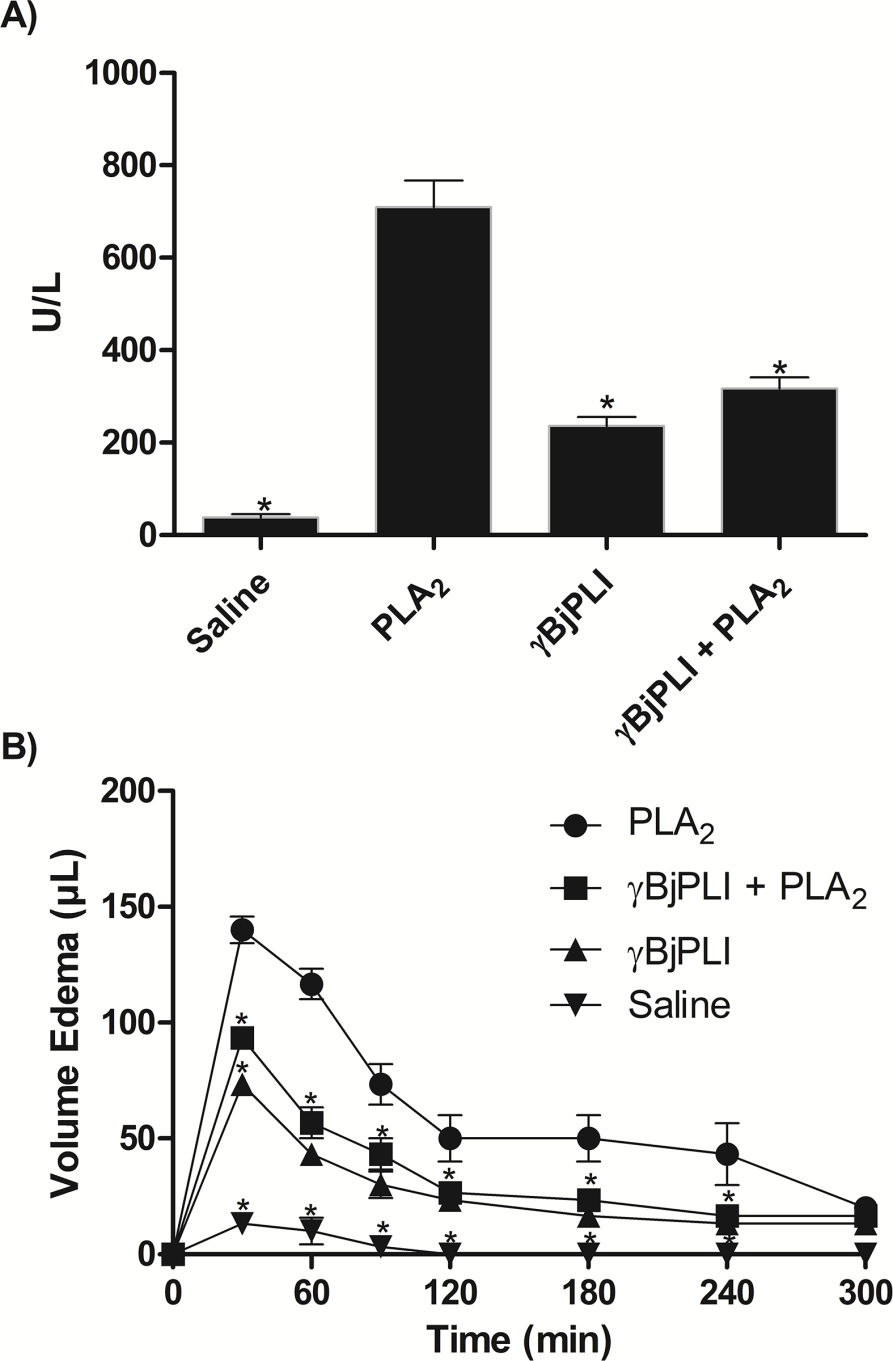
Biological activity of γBjPLI (*in vivo*). A) Neutralization of the myonecrotic activity of γBjPLI caused by PLA_2_. Samples of saline (20 μL), PLA_2_ (20 μg), γBjPLI (20 μg) or PLA_2_ + γBjPLI (20 μg + 20 μg) were preincubated for 30 min at 37°C, injected into the *gastrocnemius* muscle and then the CK present in the mice blood (Swiss) was quantified and expressed in U/L. B) Neutralization of edematogenic activity of PLA_2_. Samples of PLA_2_ (10 μg), γBjPLI (10 μg) or PLA_2_ + γBjPLI (10 μg + 10 μg), previously incubated for 30 min at 37°C, were injected into the right sub plantar region of the mice paw (Swiss). The volume of edema was monitored using a hydroplethysmometer, until the decrease of the inflammation reached 20% of the initial one.

After confirmation of *in vitro* inhibition, *in vivo* tests (for evaluation of edema and myonecrosis inhibition) were performed. The γBjPLI was able to significantly decrease both the edema and the myonecrotic effect of PLA_2_. In Fig 4A and 4B, it is possible to note that γBjPLI induced marginal myonecrosis as well as marginal edema, but after being preincubated with PLA_2_ it showed a great decrease in the damage caused by PLA_2_.

## 4. Discussion

Although PLIs have been described and purified in several species of snakes [25,51–53], *B*. *jararaca* PLI has only been reported through cDNA transcription of liver cells [32]. Therefore, this work showed for the first time, to the best of our knowledge, the isolation of a γPLI from *B*. *jararaca* serum. After two chromatographic steps, γBjPLI was isolated with a recovery of 1% of the serum proteins applied initially, which was lower than that recovered by purification of γCdcPLI (2.60%) from plasma of *Crotalus durissus collineatus*, possibly due to methodological differences and biological characteristics [34]. Variation in the levels of inhibitors of venom components found in snake plasmas is still not well understood, and may be a physiological response of the snakes to repeatedly contact with the venom or be under a genetically programmed control [54]. It is known that newborns of *Clelia clelia* (an ophiophagus snake), even without any contact with venom, have antihemorrhagic properties in serum [55]. On the other hand, recently, a study described the clear ontogenetic difference of inhibitors expression by qPCR profile analysis, in which γ-PLI had an up-regulation around 30-fold in adults in relation to juvenile of *B*. *jararaca* specimens [54]. Also, Kinkawa et al. demonstrated that the gene expression of α-PLI and β-PLI in *Gloydius brevicaudus* liver is increased by the intramuscular injection of the PLA_2_ derived from its own venom [56].

γBjPLI was found as monomers of 20 and 25 kDa (under non-reduced and reduced conditions, respectively). This molecular mass corroborates the data described by Estevão-Costa et al. [32], obtained by primary structure deduction of transcribed **γ**PLI in *B*. *jararaca*. However, native γPLI is usually found as oligomers formed by identical subunits of monomers, as PIP (γPLI from Asian python—*Malayopython reticulatus*) and BmjMIP (γPLI from *B*. *moojeni*) [31,57]. Soares et al. suggest that the oligomeric form of BmjMIP can be monomerized when occur an interaction with PLA_2_, and each subunit can bind and inactivate a myotoxin molecule [31]. So, the affinity purification process may have caused the monomerization of γBjPLI, explaining the result presented in SDS-PAGE.

The difference found in the mass values under different conditions (with and without β-mercaptoethanol) was also reported by Soares et al. [31], who suggests that the oligomers of these proteins are stabilized through non-covalent interactions, and that each subunit has internal disulfide bonds, explaining the fact that migration has been hampered by protein linearization (under reduced conditions). By contrast, under non-reduced conditions the folding is maintained, facilitating gel migration.

After preincubation of the inhibitor with PLA_2_, the interaction between the two molecules is shown by SEC, since they appear as a single fraction of about 35 kDa, which is not maintained in the RP-HPLC, suggesting that the molecular complex stability was maintained by weak molecular interaction and involves the hydrophobic interaction, easily disrupted by RP-HPLC conditions.

**Fig 5:**
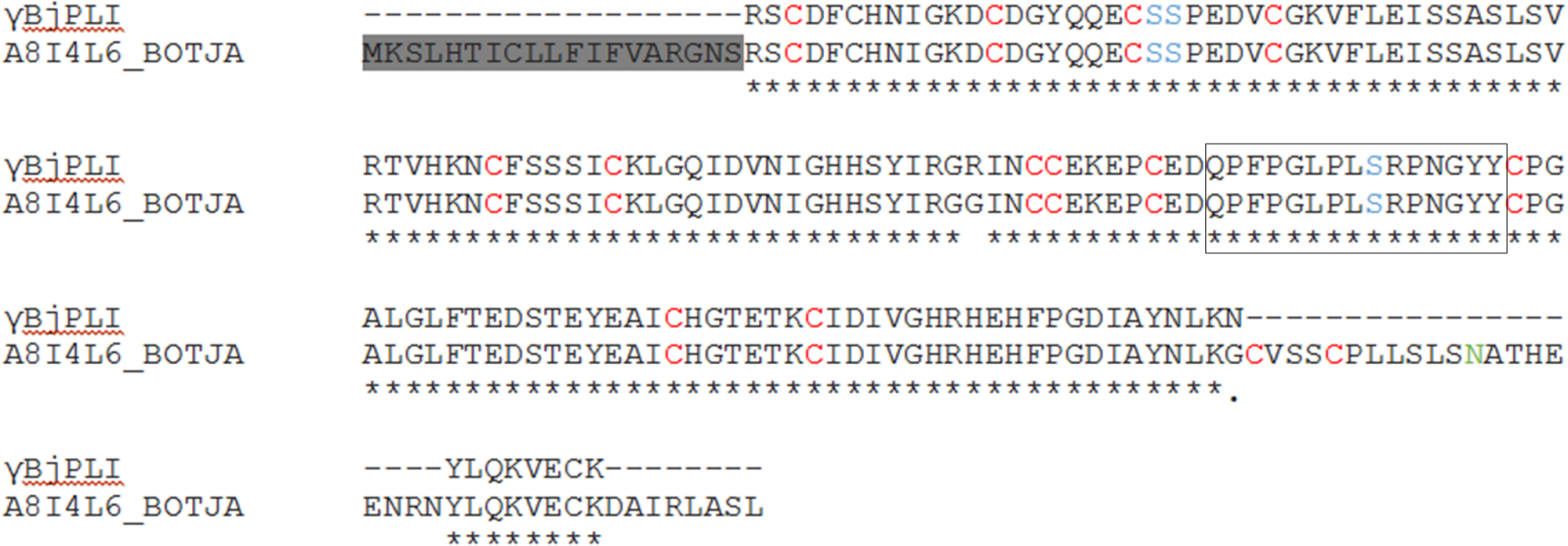
Alignment of the primary structure of γBjPLI and A8I4L6_BOTJA. Alignment of the γPLI amino acid sequence of *B*. *jararaca* (A8I4L6_BOTJA) with the peptides acquired by mass spectrometry (γBjPLI). The 16 Cys are in red, the 3 possible phosphorylation sites are indicated in blue and the N-glycosylation site is indicated in green. The oligopeptide ^104^QPFPGLPLSRPNGYY^118^ is surrounded by a rectangle. The symbol (*) indicates conserved residues; (.) indicates semi-conservative mutations. The signal peptide is highlighted in gray.

The protein eluted by affinity chromatography was only identified after trypsin digestion, mass spectrometric analysis and comparison with the database. The γBjPLI peptide sequences obtained showed similarity with two deduced *B*. *jararaca* γPLI proteins, A8I4L6_BOTJA and A8I4M0_BOTJA, with 72% and 62% coverage, respectively. In addition, another *Bothrops* snake, *B*. *moojeni*, also presented a γPLI protein (A8I4N5_BOTMO) with 42% of coverage in relation to γBjPLI [32]. When sequences of γBjPLI and A8I4L6_BOTJA were aligned (Fig 5), disregarding the signal peptide sequence, we could note that the coverage increased to 85% and that both had high similarity. The partial sequence of γBjPLI showed two substitutions: an arginine (γBjPLI) by a glycine (A8I4L6_BOTJA) at position 71, and an asparagine (γBjPLI) by a glycine (A8I4L6_BOTJA) at position 145. The region with the possible N-glycosylation site was not sequenced, probably this site should be found at position 176, since the asparagine-alanine-threonine appears in A8I4L6_BOTJA and it is a well-conserved sequence among other γPLI [32,34].

Secondary structures were analyzed by CD and showed the presence of alpha-helices (26.3%), random structures (34.5%) and beta sheets (18.5%), corroborating the analysis of Estevão-Costa [32]. *Bothrops* genera γPLIs have the same composition of secondary structures and the alpha-helix region was consistently described near to the carboxy-terminus (positions 157–164), according to the prediction by Estevão-Costa et al. [32].

The interaction site of γPLI and PLA_2_ has been suggested to be the oligopeptide ^104^QPFPGLPLSRPNGYY^118^ [32], and this sequence of amino acids was identified in γBjPLI. Some authors suggest that there may be three phosphorylation sites, at position 21 (Ser), 22 (Ser) and 111 (Thr), and that these sites may help the molecule to unleash other physiological roles, besides the inactivation of PLA_2_; however, until the moment, no other role was attributed to PLIs [32,58].

In order to evaluate the inhibition of PLA_2_ activity using γBjPLI protein, it was necessary to separate the basic fraction (PLA_2_) of crotoxin from crotapotin, which decreases the enzymatic activity of this complex [59].

Thus, isolated PLA_2_ was incubated with different concentrations of γBjPLI and analyzed using the synthetic substrate 4N3OBA. Such experiment showed the inhibitory action of γBjPLI on PLA_2_ enzymatic activity in a dose dependent manner. The same result had already been reported before and showed 46% inhibition of *C*. *d*. *terrificus* PLA_2_ by γBjussuMIP, a γPLI purified from *B*. *jararacussu* plasma [58]. Since γBjPLI was able to inhibit phospholipase activity *in vitro*, it was decided to analyze whether it also occurred *in vivo*.

It is possible to note that there was an increase in plasma CK activity and paw edema caused by γBjPLI, when compared to normal levels in controls, and similar results were showed by Gimenes et al. [34]. A question that arose from this observation was if these increases in plasma CK levels and paw edema may be due to a possible contamination with crotoxin during the isolation of γBjPLI by affinity chromatography. However, chromatographic profiles obtained by size exclusion analysis and RP-HPLC showed a satisfactory purity level of γBjPLI (when applied to the column alone). In addition, it is important to point out that no peptides related to crotoxin was identified when a sample of γBjPLI was subjected to mass spectrometry analysis, confirming that there is no contamination with this molecule during the isolation of γBjPLI by affinity chromatography.

These marginal myonecrotic and edematogenic effects probably occur due to a physiological response of our experimental model, since γPLI is not a native protein of mice. However, the increase caused by PLA_2_ was significantly higher than γBjPLI, enabling the evaluation of inhibitory activity, which strongly decreases when incubated with this inhibitor.

Thus, the paw edema test was performed in mice and γBjPLI was able to significantly decrease (~ 40%) edema caused by PLA_2_. Considering that PLA_2_ from crotoxin is an Asp49-type, it is possible that γBjPLI has a different affinity for Lys49-like PLA_2_ [60]. A similar effect was observed by Oliveira et al. [24], in which γBjussuMIP was able to inhibit more efficiently the edematogenic activity of Asp49-like PLA_2_ (77–88%) than Lys49-type (45–50%). By contrast, αBjussuMIP was more efficient inhibiting Lys49-type (91–93%) than Asp49-type (45%) [46]. The γBjPLI also inhibited the myonecrotic activity of PLA_2_, reducing ~ 61% of the damage caused by PLA_2_ in cells. In addition, γCdcPLI inhibited 27% of PLA_2_ BnSP-7 (Lys49-type PLA_2_, from *Bothrops pauloensis*) [34].

In summary, the results obtained in this work showed the isolation of a γPLI from *B*. *jararaca* serum, which can represent a new perspective for the treatment of local effects caused by *Bothrops* envenomation, since local effects are poorly neutralized by antibothropic serum.

## Supporting information

S1 Table The complete peptide table obtained from the band excised from SDS-PAGE and analyzed by mass spectrometry in Synapt G2 HDMS (Waters). **Peptide**: The amino acid sequence of the peptide as determined in PEAKS Search. A modified residue is followed by a pair of parentheses enclosing the modification mass. -**10lgP**: Peptide -10lgP score. The score indicates the scoring significance of a peptide-spectrum match. **Mass**: Monoisotopic mass of the peptide. **ppm**: Precursor mass error, calculated as 106 × (precursor mass—peptide mass) / peptide mass. **m/z**: Precursor mass-to-charge ratio. **Scan**: Scan number. **Start**: Shows the peptide's starting position in the protein. **End**: Shows the peptide's ending position (inclusive) in the protein. **#Spec**: Number of spectra assigned to the peptide. **PTM**: Types and numbers of modifications present in the peptide.
(XLSX)
